# Antagonism within mutualism: host control of symbionts through nodule-specific antimicrobial peptides

**DOI:** 10.3389/fmicb.2025.1622262

**Published:** 2025-09-11

**Authors:** Ashton A. Eaker, Shawna L. Rowe, Maren L. Friesen

**Affiliations:** Department of Plant Pathology, Washington State University, Pullman, WA, United States

**Keywords:** symbiosis, legume–rhizobia, mutualism and antagonism, plant peptides, antimicrobial peptides, gene family evolution

## Abstract

Legumes (Fabaceae) have developed a symbiotic relationship with nitrogen-fixing bacteria called rhizobia to meet their nitrogen needs. Legumes recruit rhizobia from the soil, house them in root organs called nodules, and manipulate bacterial metabolism, providing carbon and receiving bacterially fixed nitrogen in return. One mechanism of host control is through a family of antimicrobial peptides that only appears in the inverted repeat lacking clade (IRLC) of the legumes, though the Dalbergioid clade has similar peptides. They are named nodule-specific cysteine-rich (NCR) peptides due to their exclusive expression in the nodule during symbiosis and the shared 4 or 6 cysteine residue motif. These genes and subsequent proteins vary in number, sequence, and function, but evolutionary genomics research shows that they are adapted from the plant immune system for the new function of symbiont manipulation. In this review, we present the current understanding of NCR peptide biology, expression, and function. We examine NCR genomic and biochemical features and explore their roles in shaping symbiotic outcomes. Finally, we discuss emerging applications and key open questions. Understanding host manipulation of bacterial symbionts within plant tissues provides researchers with targets for engineering more efficient nitrogen-fixing symbioses. In addition, NCR peptides show promise as therapeutic agents with the potential to control both plant and animal pathogens.

## 1 Introduction—antimicrobial peptides in symbiotic relationships

**Figure 1:**
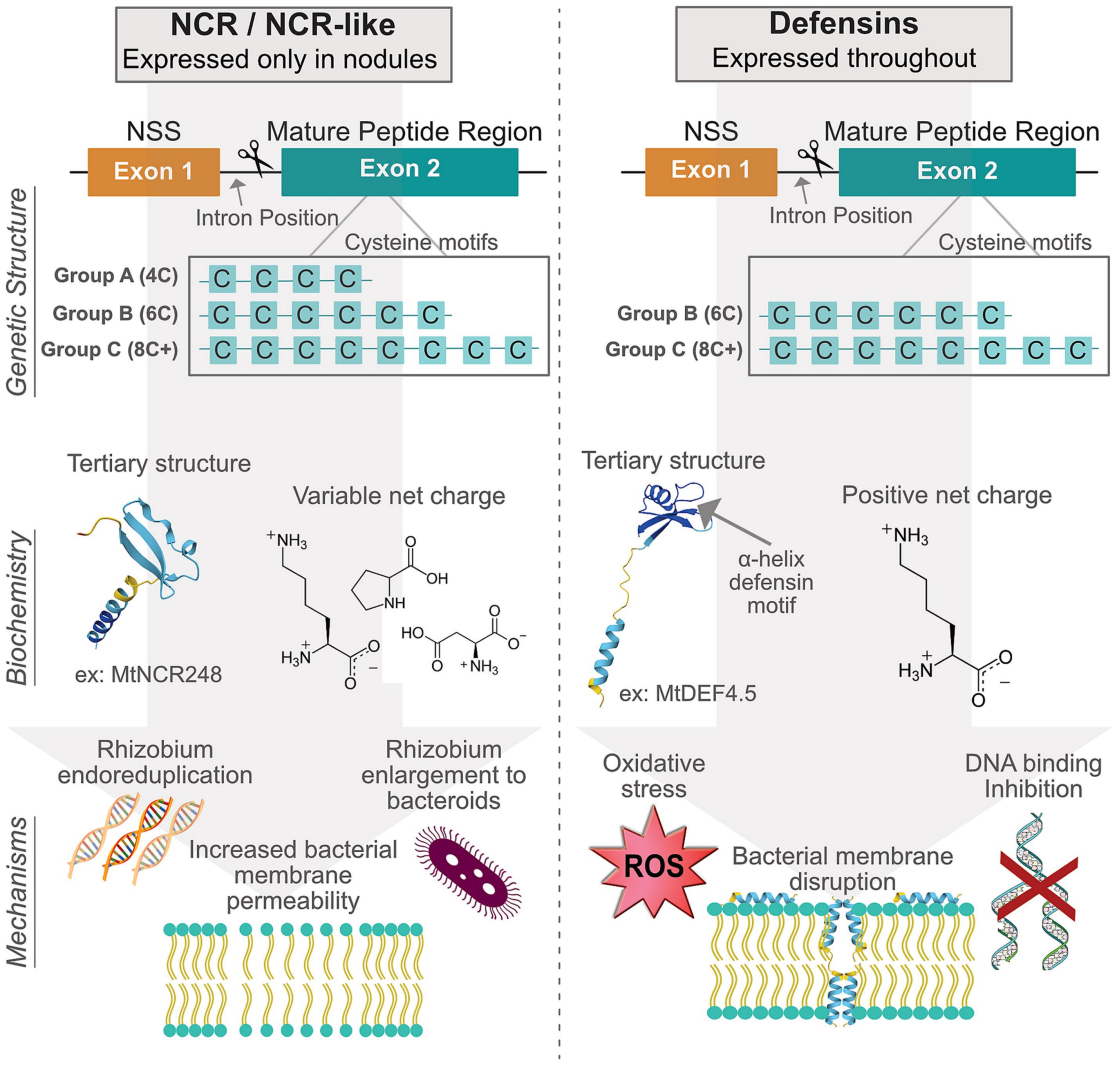
Comparison between NCR/NCR-like peptides and more general antimicrobial peptides (defensins). The left side of the figure illustrates NCR/NCR-like features, while the right side shows defensin features. At the top are schematic gene architectures highlighting conserved exons for the N-terminal secretion sequence and the mature peptide region with their characteristic cysteine motif groups. The middle section presents biochemical features, including variable net charges with example amino acid residues, together with representative tertiary structures of MtNCR248 (NCR/NCR-like) and MtDEF4.5 (defensin). The bottom depicts three mechanisms of action for NCR/NCR-like peptides and defensins.

Many plants deploy cysteine-rich antimicrobial peptides (AMPs) as a first line of defense against pathogens. A specialized subset of these peptides is produced only in the root nodules of leguminous plants, earning them the name nodule-specific cysteine-rich, or NCR, peptides. NCRs evolved via duplication of ancestral defensin-like AMPs and retain the hallmark 4–6 conserved cysteines (occasionally 8–10) arranged to form disulfide bonds, their characteristic stabilizing tertiary structure (Figure 1; Mergaert et al., 2003).

**Figure 2:**
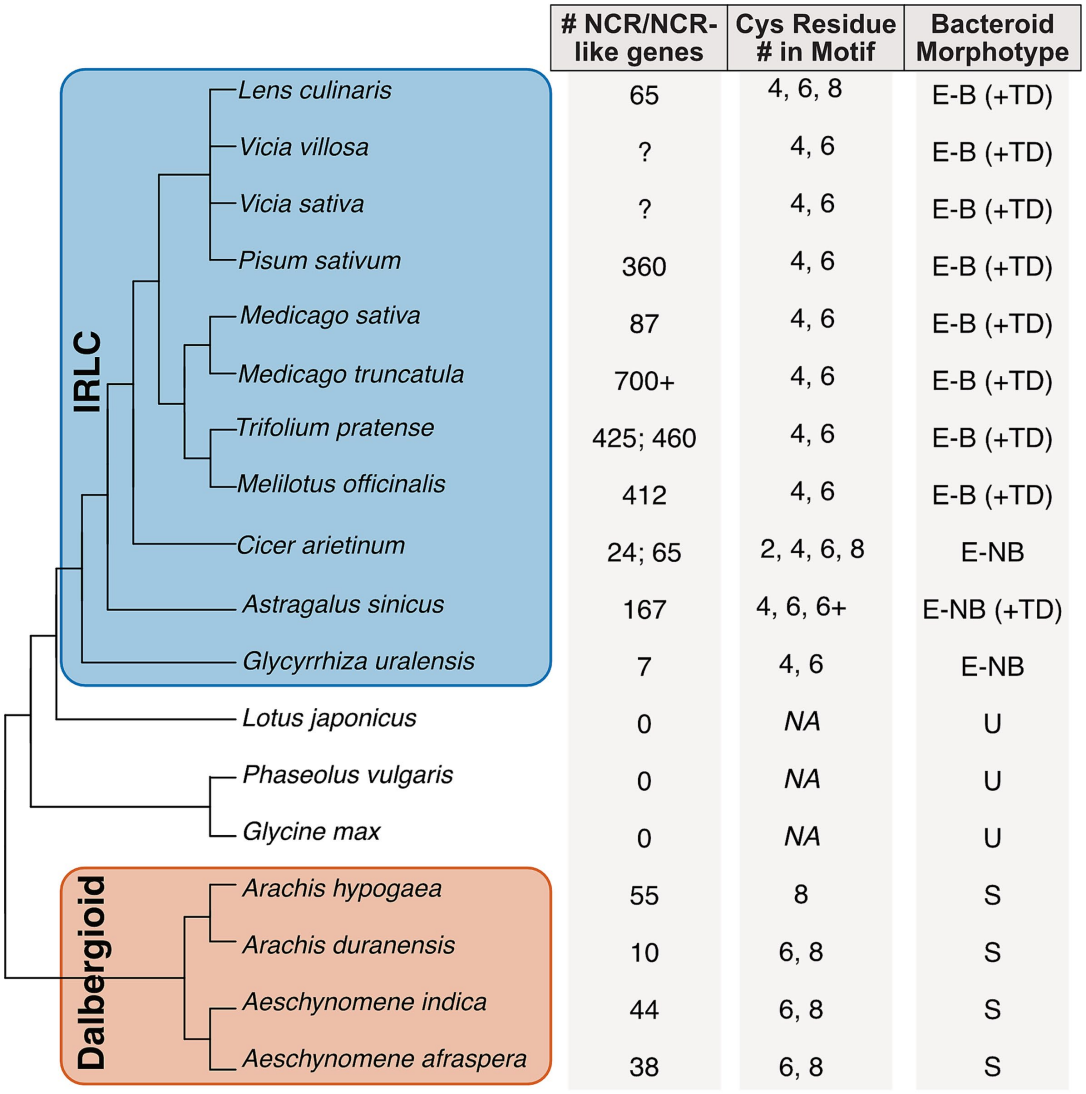
Phylogenetic distribution and gene characteristics of NCR and NCR-like peptides in legume species. Phylogenetic tree of 18 selected legume species (Papilionoideae) highlighting the IRLC and Dalbergioid clades. To the right of the phylogeny columns show (1) NCR/NCR-like gene counts. If none were reported by the authors, a “?” Is listed. If two values have been published, both are shown. (2) Total number of cysteine residues present in the motif structure. NA indicates no motif, and (3) corresponding bacteroid morphotypes present in host nodules. E-B ± TD: elongated-branched ± Terminally Differentiated; E-NB ± TD: elongated non-branched ± TD, U: unmodified; S: spherical.

To date, NCRs have only been identified in the informal inverted repeat-lacking clade. The IRLC—a monophyletic group that counts the medics, peas, and clovers among its members (Figure 2)—uniquely produces NCRs (Guefrachi et al., 2014). The model legume, *Medicago truncatula*, is a member of the IRLC and is where the NCR gene family was first characterized (Mergaert et al., 2003). Although NCRs are antimicrobial *in vitro*, they induce significant physiological change in the *M. truncatula* rhizobial symbiont, *Ensifer meliloti*, when studied *in planta.* All rhizobia show altered physiology within nodule cells in which they transform into nitrogen-fixing, organelle-like structures called bacteroids (Peña et al., 2018). Individual bacteroids become enveloped within a plant-derived membrane, collectively known as the symbiosome, where a host provides an optimal microaerobic environment to protect oxygen-sensitive nitrogenases and delineate a zone of control for the transactional exchanges of host carbon and symbiont nitrogen. NCR activity in the nodules helps orchestrate the processes that underpin these features by deploying modified antimicrobial mechanisms that alter bacteroid physiology (Mergaert, 2020).

**Figure 3:**
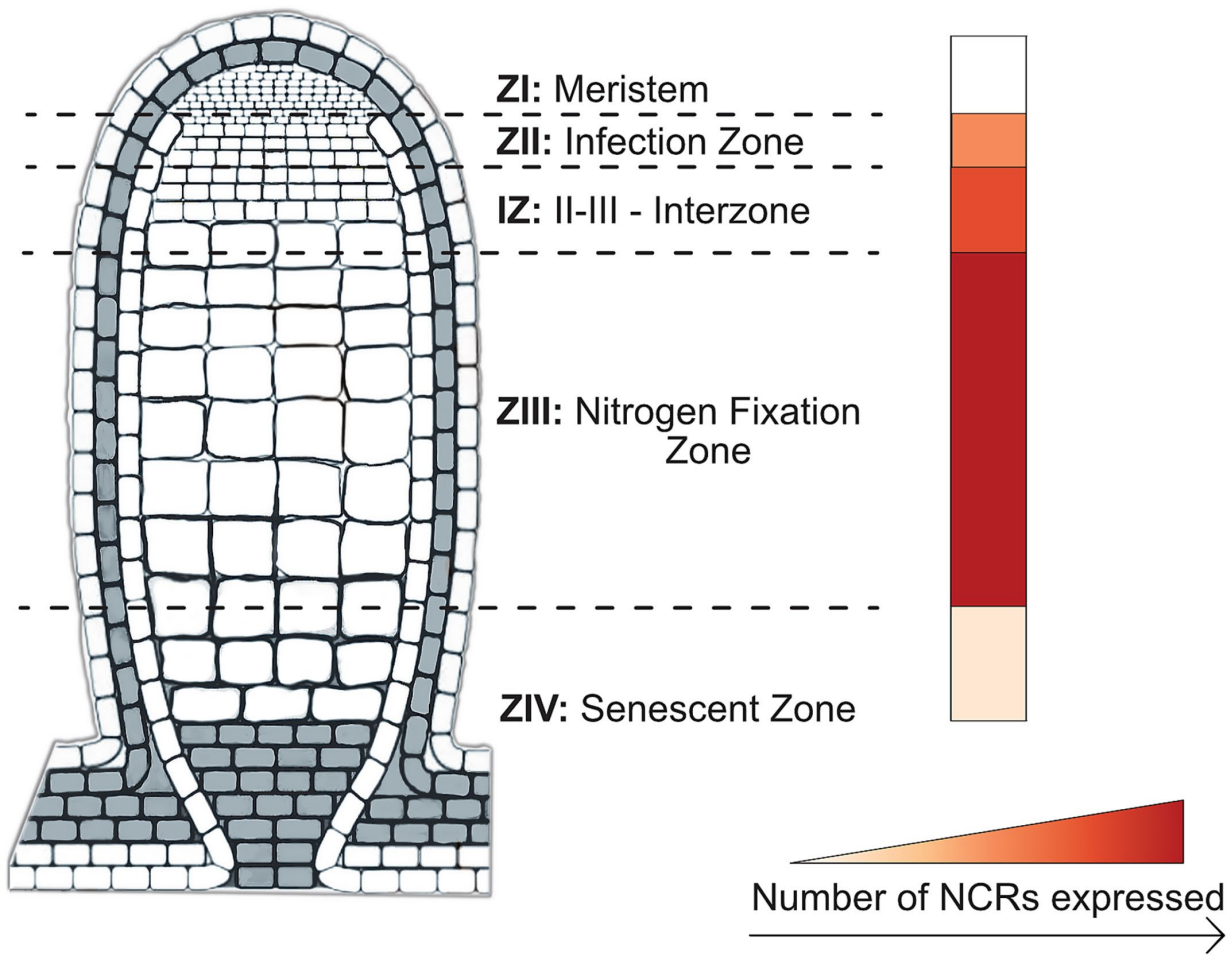
Spatial mapping of the relative quantity of NCRs expressed at 4 weeks post-nodulation in *M. truncatula* nodules. Schematic of an *M. truncatula* root nodule delineated into zones ZI-ZIV (ZI: meristem; ZII: infection zone; IZ: interzone; ZIII: nitrogen fixation zone; ZIV: senescent zone), accompanied by a heat map bar showing greatest abundance of expressed NCRs at 4 weeks. Highest (darkest) to lowest (lightest) in order are: ZIII, IZ, ZII, ZIV, and ZI. Visual representation of nodule was adapted from Lepetit and Brouquisse (2023), licensed under CC BY.

IRLC legumes form elongated root nodules, known as indeterminate nodules, characterized by a persistent meristem and defined zones that enable continuous growth (Figure 3). Indeterminate nodules, in contrast to the spherical determinate nodules of non-IRLC species like *Glycine max* (soybean) and *Lotus japonicus*, have the capacity to induce terminal differentiation in rhizobia—an irreversible process rendering the former free-living bacteria an enlarged, organelle-like cell unable to persist beyond their host (Haag and Mergaert, 2019). While all IRLC species develop indeterminate nodules, only those producing NCR peptides induce terminal differentiation of bacteroids within symbiosomes (Montiel et al., 2016). In addition to terminal differentiation of bacteroids, NCRs also induce cell differentiation in indeterminate nodule types, giving rise to spatially and functionally distinct nodule zones (Figure 3). Interestingly, recent findings have revealed an alternative mechanism for cell differentiation independent of NCRs with the discovery of temporary nodule zonation in determinate nodules. The observed cell-type differentiation was found to be modulated by NIN2a-GH3-mediated auxin conjugation in determinate soybean nodules, though the differentiation largely disappears during nodule maturation and leaves the inhabiting rhizobia reproductively viable (Tu et al., 2024). NCR-induced bacterial reprogramming irreversibly transforms rhizobia into one of two terminal bacteroid morphotypes: elongated (E-type) or spherical (S-type) cells, both having suppressed cell division and amplified genomes (Montiel et al., 2016, 2017). Terminal differentiation likely provides superior host benefits through more favorable C:N exchange rates. Rhizobia, in turn, have mechanisms that seem to counteract some of the effects of NCRs; this has led to the “working balance” hypothesis, wherein the host plant needs to have enough NCRs of the appropriate type to overcome rhizobial defenses, but not so many NCRs that the rhizobia are destroyed (Pan and Wang, 2017; Wendlandt et al., 2022). Taken together, NCRs function to facilitate symbiosis by enabling host domestication of their bacterial symbionts rather than their extermination.

To date, only a handful of the more than 700 NCR peptides identified in *M. truncatula* have been characterized, with many being essential for a successful symbiotic nitrogen fixation (Table 1). NCRs have been discovered and partially characterized in other IRLC legumes, and NCR-like peptides that give rise to similar symbiotic features have been identified in the Fabaceae clade, Dalbergioid (Czernic et al., 2015; Nicoud et al., 2021). Although characterized peptides represent only a small fraction of the known diversity, accumulating evidence clearly suggests these peptides have critical roles in host-mediated regulation of the legume–rhizobia symbiosis.

**Table 1:** NCRs essential to symbiosis.

NCR ID	pI	Mature peptide length (aa)	Cysteine motif	Molecular function	Reference
MtNCR169	8.45	38	4	Binds phospholipids	Isozumi et al. (2021)
MtNCR211	5.38	34	4	Blocks colony formation of free living rhizobia	Kim et al. (2015)
MtNCR247	10.15	24	4	Binds heme, interacts with ribosomal proteins, FtZ, interacts with inner and outer membrane components	Farkas et al. (2014), Jenei et al. (2020) and Sankari et al. (2022)
MtNCR044	10.32	36	4	Membrane permeabilization, elicits reactive oxygen species production, interacts with ribosomal proteins	Velivelli et al. (2020)
MtNCR343	6.34	43	4	Localizes to symbiosome, accumulates in the peribacteroid space, maintains bacteroid viability, and reduces bacteroid stress response	Gao et al. (2023) and Horváth et al. (2023)
MtNCRnew35	4.78	47	4	Localizes to the symbiosome	Horváth et al. (2023)
MtNCR086 and MtNCR314 (duplicate)	8.66	34	4	Essential for bacteroid differentiation	Saifi et al. (2024)

In this review, we present the current state of knowledge regarding the protein biology and expression of NCR peptides across both model and non-model legume species. Next, we describe a recent study that sheds light on extremely rapid gene family evolution of these antimicrobial peptides within both IRLC and non-IRLC legumes. We then present insights on the regulation of NCRs by both the legume host and rhizobial symbiont. Finally, we discuss emerging applications translating NCRs into weapons against pathogens and highlight key open questions.

## 2 NCR peptide biology

### 2.1 Evolutionary origins and structural features of NCRs

Evolutionary evidence of the NCR gene family diverging from AMPs can first be found in their genetic structure. NCRs, their relatives in Dalbergioid nodules, and defensins share a remarkably conserved gene structure despite significant sequence diversity around the key cysteine motifs (Figures 1A,B; Salgado et al., 2022). NCRs and similar genes usually contain two exons separated by a single intron consistently placed between nucleotides 1 and 2 of a codon, a few triplets before the first cysteine codon. Rarely, genes of this form possess an additional exon; even so, the functional separation of the two exons is also highly conserved, with the first generally encoding a small signal peptide while the second encodes the mature peptide containing the hallmark cysteine residues (Mergaert et al., 2003).

Cysteine, an amino acid with a thiol functional group, is able to form covalent linkages with nearby thiol residues to form disulfide bridges. The strong, stabilizing bonds form between pairs of cysteine residues, so the conserved motif forms are multiples of 2 and are sometimes categorized by their specific motif structure. Group A has the 4 cysteine motif, group B has the 6 cysteine motif, and group C has more than 6 cysteine residues. Most NCR and NCR-like genes fall in groups A and B, having the same structure of MtNCRs (Figure 1A), and defensins tend to be group C motifs (Figure 1B; Mergaert et al., 2003; Li et al., 2016; Ma et al., 2023). All motif groups result in peptides with a highly conserved tertiary structure that sets the stage for amazing diversity and symbiotic functionality.

### 2.2 Biochemical properties and diversity of NCRs

Beyond conserved gene and peptide structures, NCRs, Dalbergioid analogues, and defensins can be distinguished by key physicochemical properties that directly influence their biochemical functions (Figures 1A,B). All forms are small, ranging from 24 to 67 amino acids in length, with NCRs trending smaller than defensins (Maróti et al., 2015). Unlike the predominantly cationic defensins, NCR net charges range from strongly anionic (pI < 4.5) to strongly cationic (pI > 9.5). This charge variability correlates with mechanistic diversity; cationic NCRs (e.g., MtNCR247, MtNCR335) demonstrate membrane-disruptive activities analogous to defensins, while anionic NCRs (e.g., MtNCR169) facilitate iron transport and metabolism (Isozumi et al., 2021). The tertiary structures of NCRs lack a characteristic α-helix defensin motif, instead featuring a β-sheet arrangement in the C-terminus region with a largely unstructured N-terminus. This structural distinction enables NCRs to interact with diverse bacterial targets, from membrane phospholipids to cytoplasmic proteins like ribosomes. Hydrophobicity patterns also differ significantly; defensins maintain amphipathic surfaces critical for microbial membrane insertion, whereas NCR and NCR-like peptides display variable hydrophobic patches that influence their diverse functional features (Yount and Yeaman, 2004; Velivelli et al., 2020).

Outside of the IRLC, a recent study has expanded on our understanding of the evolution of this gene family and has described “NCR-like” peptides in the Dalbergioid clade (Figure 2; Czernic et al., 2015; Nicoud et al., 2021; Raul et al., 2022). These NCR-like genes show similar conservation of the cysteine motifs and tertiary structure, but studies have found sequence differences that suggest an evolutionary split from the NCRs. Intriguingly, plants with identified NCR-like peptides in Dalbergioid form a type of nodule that is neither indeterminate nor determinate; instead, their nodules appear as a sort of hybrid between the two main types but result in terminally differentiated bacteroids (Mergaert, 2020). This finding further suggests that NCRs play a key role in the formation of terminally differentiated bacteroids and a seemingly elevated degree of host control over the inhabiting rhizobia. The key features of NCR and NCR-like peptides are summarized in Figure 1.

## 3 NCR gene expression and regulation

### 3.1 Expression patterns of NCRs in model systems

A recent study has expanded our understanding of the core NCR gene structure (Figure 1), the extent and diversity of the gene family, and transcriptional regulation both in and outside of the model plant, *M. truncatula*. Expression analyses now include work in multiple nodule types, across nodule zones, through developmental time, and when challenged by alternative or mutant symbionts.

#### 3.1.1 Essential NCRs for symbiosis

First identified in legumes of the informal IRLC, the majority of what is known regarding the identity and mechanisms of NCRs comes from work in *M. truncatula*. When first discovered, the gene family was believed to contain around 300 members (Mergaert et al., 2003); as of 2023, the estimate is over 700, with a growing number determined as necessary for symbiosis in *M. truncatula* (Branca et al., 2011; Young and Bharti, 2012; Zhang et al., 2023). In a recent reverse genetic study, researchers interrogated individual MtNCR genes and gene products for their necessity or sufficiency in maintaining a healthy symbiosis (Horváth et al., 2023). In generated mutant lines, researchers identified MtNCR343 and MtNCR-new35 as the knocked-out genes responsible for an ineffective symbiosis phenotype. Subsequently, forward genetic screening with a GUS reporter–promoter fusion with these genes showed that MtNCR343 is expressed in the infection zone (ZII) and nitrogen-fixing zone (ZIII), and MtNCR-new35 in ZII, but at much lower transcript abundance (see zones in Figure 3). Despite the lower transcript abundance of MtNCR-new35, the peptide product was abundant in ZII, which the authors state is evidence for slow turnover or enhanced stability of the MtNCR-new35 peptide. Additionally, the fluorescently labeled and functional mature peptides of MtNCR343 and MtNCR-new35 colocalized together at the bacteroid membrane and were able to rescue the ineffective symbiosis phenotype (Horváth et al., 2023).

Some characterized NCRs are not required for symbiosis but are involved in optimizing symbiotic functioning. A recent study (Saifi et al., 2024) investigated an *M. truncatula* symbiotic mutant (FN9285) that was defective in its ability to initiate terminal differentiation of the free-living rhizobia to the bacteroid state inside nodules. The causative mutation was determined to be the deletion of a cluster of 9 NCR genes: MtNCR086, MtNCR087, MtNCR132, MtNCR136, MtNCR165, MtNCR301, MtNCR314, MtNCR406, and MtNCR583. All nine were peptides containing the Group A 4 cysteine motif. The mature peptide products of MtNCR086 and MtNCR314 are identical, with the only difference between the genes being two amino acid substitutions in the signal peptide. To further narrow down the causative deletion, the researchers complemented FN9285 with each MtNCR separately in an attempt to rescue the defective phenotype. The only transformations that showed functional nodules and a healthy green plant were MtNCR086 and MtNCR314. Targeted mutation of MtNCR086, MtNCR314, and MtNCR583 further showed that MtNCR086 and MtNCR314 are required for healthy symbiosis, while MtNCR583 is not necessary. The primary difference between MtNCR086 and MtNCR314 was a higher transcription of MtNCR086 (about two orders of magnitude); despite this difference, MtNCR314 could still rescue the defective phenotype in the absence of MtNCR086. The promoter activity of both genes was similar, suggesting that some other regulatory element is responsible for the lower transcript abundance of MtNCR314. This study adds to the list of NCRs required for a functional symbiosis between *M. truncatula* and *E. meliloti*. When grouped with other essential NCRs, there is no obvious pattern in expression, exon number, or isoelectric point (pI) that determines a functional symbiotic outcome, further supporting the model of NCRs as a mechanism with diverse outcomes meant to optimize the symbiosis (Saifi et al., 2024).

#### 3.1.2 Temporal and circadian regulation of NCRs

Temporal factors strongly influence the expression patterns of at least some MtNCR genes. New evidence suggests that at least 45 NCRs in *M. truncatula* are under circadian rhythm control (Achom et al., 2022). It was found that the transcription of these NCRs peaks in the evening and late night/early morning. This rhythmic transcription pattern suggests an adaptation by the plant host to synchronize nitrogen fixation, an energy-intensive process, with dark periods to utilize reserves accumulated from photosynthesis. Other time-course studies have shed light on MtNCR expression patterns across nodule developmental stages. During nodule senescence, 454 out of 700 MtNCRs are downregulated alongside MtDME, a DNA demethylase known to regulate NCR gene expression. Additionally, no NCR associated with bacteroid differentiation was upregulated during senescence (Sauviac et al., 2022). A similar pattern of widespread NCR downregulation has also been observed in disrupted symbiosis systems—a finding that logically fits with the senescence findings (Moore et al., 2021). Collectively, these findings reinforce a model in which the host legume actively modulates NCR expression to control bacteroid differentiation and nitrogen fixation in response to developmental and environmental cues.

### 3.2 Expression patterns of NCRs in non-model legumes

Investigation of the NCR gene family and peptides outside of *M. truncatula* has uncovered diversity within legumes that produce canonical NCRs and the existence of NCR-like antimicrobial peptides produced outside of the IRLC. Studies have successfully leveraged the transcriptomics of the nodule to describe and quantify gene family expression and regulation in contrasting symbiotic contexts (see Table 2).

**Table 2:** NCR or NCR-like genes identified across legumes.

Species (common name)	IRLC?	Gene count	Cysteine motifs	Reference
*Aeschynomene afraspera* (African jointvetch)	No	38	6, 8	Czernic et al. (2015)
*Aeschynomene indica* (Indian jointvetch)	No	44	6, 8	Czernic et al. (2015)
*Arachis hypogaea* (peanut)	No	55	8	Raul et al. (2022)
*Astragalus sinicus* (Chinese milk vetch)	Yes	167	4, 6, 6+	Wei et al. (2022)
*Cicer arietinum* (chickpea)	Yes	24;65	2, 4, 6, 8	Kant et al. (2016) and Montiel et al. (2017)
*Lens culinaris* (Lentil)	Yes	65	4, 6, 8	Durán et al. (2021)
*Medicago sativa* cv. Algonquin (alfalfa)	Yes	308	4, 6	Huang et al. (2022)
*Medicago sativa* G3 (alfalfa)	Yes	87 (Differentially expressed)	4, 6	Kang et al. (2020, 2022)
*Medicago sativa* G9 (alfalfa)	Yes	87 (Differentially expressed)	4, 6	Kang et al. (2020, 2022)
*Medicago truncatula* (barrel medic)	Yes	700	4, 6	Pecrix et al. (2018)
*Melilotus officinalis* (yellow sweet clover)	Yes	412	4, 6	Huang et al. (2022)
*Pisum sativum* L (pea)	Yes	360	4, 6	Zorin et al. (2022)
*Trifolium pratense* L (red clover)	Yes	425;460	4, 6	Trujillo et al. (2019) and Dinkins et al. (2022)

#### 3.2.1 Transcriptomic diversity across species expressing NCRs

In *Trifolium pratense* (red clover), an important forage legume in pastures, Dinkins et al. (2022) compared gene transcription in roots and nodules of inoculated and uninoculated plants. They identified 460 putative NCRs in the *T. pratense* genome (Tpv2.1) and were able to confirm the expression of 425 in their study. The majority of the TpNCR genes followed the canonical structure of two exons interrupted with one intron near the signal peptide cleavage site (Figure 1A), but the authors also identified 32 genes that had an additional intron near the stop codon on the 3′ end of the gene. They also annotated 18 TpNCRs that do not contain introns. *T. pratense* has a notably higher proportion of cationic peptides, 32%, compared to *M. truncatula* at ~15%. This variation in charge and subsequent antimicrobial activity could be indicative of the strategy each legume species uses to control its symbionts.

Transcriptomic analyses on nodules of *Melilotus officinalis* and *Medicago sativa* revealed that NCR transcripts accounted for roughly 9% of all detected nodule transcripts in both species (Huang et al., 2022). The authors identified 412 NCRs in *M. officinalis* and 308 in *M. sativa*. Curiously, when comparing *M. officinalis* and *M. sativa* nodule transcriptomes to *M. truncatula*, they found that only 40 NCRs were shared between the three species. Of the 40, they could not identify the two well-studied NCRs known to be essential for symbiosis in *M. truncatula* (MtNCR169 or MtNCR211). These results indicate that NCRs are undergoing rapid gene family evolution across closely related leguminous species.

#### 3.2.2 Species-specific NCR expression patterns

An investigation of the symbiotic specificity between two *M. sativa* cultivars (G9, G3) and two biotypes of the symbiont *E. meliloti* (E1, E2) showed that there is biotype-specific MsNCR gene expression in these two cultivars of *M. sativa*. G9 had high expression of 87 MsNCR genes and promoted expression of genes related to plant immunity, symbiotic specificity, nodule formation, and nitrogen fixation. In contrast, *E. meliloti* biotype had a weaker effect on these same genes in *M. sativa* G3. A gene expression network analysis was performed using only the upregulated genes associated with a high dry weight phenotype. This gene expression network identified hub node genes, which have more links than the average node, indicating a larger influence on the overall network structure. Two hubs in this network included MsNCRs, hub1, which regulated 18 other genes, and hub15 which interacted with 473 other genes. The authors conclude that the upregulation of these genes (MsNCRs, Glycine Rich Peptides, LEED…PEED, nodulins, and leghemoglobin), which work together as hubs, is what enabled G9 to negotiate a more beneficial symbiosis with its symbionts, evidenced by G9’s higher host fitness (Kang et al., 2022). Another transcriptomic study of *Pisum sativum* (common pea) nodule zones also found PsNCRs and PsNCR-like genes were prominent in the upregulated genes, particularly when comparing the early infection zone and late infection zone, as well as the early infection zone and nitrogen fixation zone. They also identified the same classes of NCR-associated genes like leghemoglobin and nodule-specific glycine-rich peptides, further emphasizing their central role in nodule maturation across legume species (Kusakin et al., 2021).

The cover crop IRLC legumes *Vicia villosa* and *Vicia sativa* have variable reported nitrogen fixation efficiencies, with *V. villosa* having more and larger nodules than *V. sativa*. To explore the molecular mechanisms underpinning the discrepancy, Ren et al. (2024) compared the two species grown in nitrogen-free and nitrogen-supplemented conditions. *V. villosa* scored significantly higher using their nitrogen response metric under nitrogen-free (94.19% higher) and nitrogen-supplemented conditions (82.91% higher). When observing bacteroid morphology with transmission electron microscopy (TEM), they found that bacteroid differentiation was ~20% higher in *V. villosa* than in *V. sativa,* while a transcriptional analysis performed on the same sample detected an enrichment of flavonoid biosynthesis genes and NCR-related genes. They attribute the increased nitrogen response in *V. villosa* to its larger biomass, larger genome size (~15% larger), larger nodule infection area, and higher proportion of bacteroids that are terminally differentiated, which could be due to the enriched expression of NCRs, though the total number of NCRs was not reported. This study supports the idea that NCRs are leveraged by the host to tighten rhizobial control and enhance symbiotic nitrogen fixation efficiency.

Wei et al. (2022) added to our understanding of NCR genes in the IRLC species, *Astragalus sinicus*. They identified 167 AsNCR transcripts that fell into the previously described cysteine motif groupings. Groups A and B were found to have the same structure as MtNCRs and an even distribution of anionic and cationic peptide products. The remarkable result of this study is that AsNCRs are dominated by group C peptides that are longer than average (>60aa) with a pI > 8 and predominantly cationic peptides (55%). This third group of NCRs falls between plant defensins and MtNCRs, but are longer with more variation in sequence outside of the cysteine motif.

### 3.3 Host-mediated regulation of NCRs

New connections have been made between NCR activity and whole plant regulatory loops as a result of advanced genomic and protein–protein interaction studies. Circadian regulation of NCRs was discovered after the circadian rhythm promoter motif was identified upstream of NCR genes; relatedly, researchers investigating genomic structure found evidence for a layer of epigenetic regulation due to the proximity of the NCR gene to transposable elements in the *M. truncatula* genome (Pecrix et al., 2018). Protein-focused studies have revealed evidence for NCR membrane binding abilities and secondary intracellular antimicrobial actions (Wu and Wang, 2019). In true form to their ancestors, both the host plant and the bacterial symbiont modulate the impact of NCRs, with the bacterial symbionts deploying methods to survive the bombardment of the antimicrobial plant peptides. Several studies use network analysis to generate hypotheses about direct and indirect consequences of NCR presence in nodules.

Recent transcriptomic evidence suggests NCRs could be a key mechanism used to sync bacterial metabolism to the plant’s circadian rhythm. Performing a time course sampling on *M. truncatula* nodules, Achom et al. (2022) isolated nodule-specific transcripts and found 45 MtNCR transcripts that were rhythmically expressed. The MtNCR transcripts were enriched in evening and night clusters of all the rhythmic transcripts. Further investigation of the MtNCR promoter regions revealed an enrichment of the Evening Element (EE) 5.4-fold compared to all rhythmic promoters. EE is a known binding site for late elongated hypocotyl (LHY) and circadian clock-associated 1 (CCA1) transcription factors. Additionally, the authors identified an alternative to EE, called Evening Element Related (EER), which was also enriched in rhythmic MtNCR promoters by 3.6-fold compared to all rhythmic transcripts. One of the primary differences between EE and EER is the introduction of a cysteine residue (AGA[T/C]ATTT) to the motif (Achom et al., 2022).

When investigating epigenetic regulation of symbiotic gene islands in the *M. truncatula* genome, Pecrix et al. (2022) found that in the root, genes associated with nodule development, differentiation, and fixation zones, and the flanking transposable elements (TEs) were methylated. DNA methylation plays a crucial part in plant development and stress responses, especially in plants with complex genomes, by modulating the access that transcriptional machinery has to a given DNA (Zhang et al., 2018). In contrast, this same set of genes, including 380 MtNCRs, leghemoglobin, symbiotic immune response regulators, redox control proteins, and long non-coding RNAs, was in hypo-differentially methylated regions (DMRs) in the nodule. Hypo-DMR reduces methylation, thereby increasing access to the DNA and the expression of transcripts encoded by the affected sequence (Zhang et al., 2018). The differences observed in methylation types and patterns imply strong epigenetic control of gene expression in the nodule across zones.

Recently, we gained the first report of a cis-acting and nodule-specific DNA-binding protein in the Type 1 AT-Hook Motif Nuclear Localized (AHL) transcription factor family that is needed for expression of the MtNCR169 gene. AHLs are conserved in non-IRLC legumes and can induce expression of MtNCR169 in non-IRLC legume nodule cells. After identifying the AHL binding motif in the promoter region of MtNCR169, the authors found the same motif in 280 other NCRs. Using the MtNCR169 promoter region as bait in a yeast 2-hybrid screen, they confirmed binding of MtAHL1, MtAHL2, GmAHL1, and GmAHL2 to the promoter in both *M. truncatula* and *G. max* nodule extract. Knocking out either MtAHL1 or MtAHL2 stopped bacteroid differentiation and resulted in nodule cells containing dead, non-elongated bacteroids (Zhang et al., 2023). The NCR gene promoters in *P. sativum* have putative binding sites for the master transcription factor TF-NLP7, which is known to regulate nitrate response through interaction with the root mobile peptide CLE-RS2 (Zorin et al., 2022). This suggests an intriguing integration of nitrogen demand signaling and symbiont regulation via NCRs.

### 3.4 Symbiont-mediated regulation of NCRs

Some bacterial partners of legumes have accessory plasmids that encode a host-range restriction peptidase (HrrP) that cleaves and inactivates NCRs during symbiosis (Price et al., 2015). Recently, researchers showed that the symbiotic partner of *M. truncatula*, *Ensifer meliloti* (previously known as *Sinorhizobium*), expresses a metallopeptidase HrrP homolog named symbiosis-associated peptidase (sap) gene whose enzymatic product SapA cleaves MtNCR035 *in vitro*. The authors propose that sapA is a cytoplasmic enzyme that inactivates MtNCRs once they have been imported into the bacteroid and their disulfide bridges have been reduced (Benedict et al., 2021). From an evolutionary perspective, the presence of *hrrP* in a wild population of *Ensifer medicae* has broad effects on both partners’ fitness, which is dependent on plant host genotype and experimental years. The fitness trait measurement and interaction with *E. medicae* strains were primarily neutral (41 out of 60), then positive (15 out of 60), with a small proportion of negative interactions (4 out of 60). Of the trait measurements between plant genotypes, the majority were consistent (14 out of 17), and the remaining were inconsistent (3 out of 17) across plant genotypes. The authors conclude that the overall positive effect that *hrrP* has on plant and symbiont fitness aligns with the working balance hypothesis between the plant NCRs and symbiont NCR tolerance mechanisms (Pan and Wang, 2017; Wendlandt et al., 2022).

Originally identified in *E. meliloti*, the *bacA* gene encodes the transmembrane domain of an ATP-binding cassette transporter system and is essential to successful symbiosis with *M. truncatula* (LeVier and Walker, 2001). There is experimental evidence that BacA is an antimicrobial peptide transporter and essential to symbiosis with IRLC legumes that produce NCRs, but dispensable when in symbiosis with non-NCR-producing legumes (Haag et al., 2012). In a recent study, Eardly et al. (2022) identified orthologs of *bacA* in a variety of *Rhizobium* species that nodulate both *Medicago sativa* (produces NCRs) and *Phaseolus vulgaris* (does not produce NCRs), but not in *Paraburkholderia* symbionts tested. During symbiosis with *M. sativa*, bacteroids of *Rhizobium* with *bacA* can survive, but they do not differentiate and have a lower nitrogen fixation efficiency than *E. meliloti*. This suggests a different sensitivity to NCRs despite having one of the tolerance mechanisms to NCRs. The specificity of the BacA transport system is hypothesized to be based on the charge and subsequent isoelectric point of the NCR peptide. When comparing NCRs in *M. sativa* and *M. officinalis*, Huang et al. (2022) found a 2.4-fold increase of cationic peptides (pI > 9.0) in *M. sativa*. These authors propose that the ability of *Rhizobium leguminosarum* with *bacA* to form successful symbiosis with *M. officinalis* and *P. sativum* but not *M. sativa* is based on the high proportion of cationic NCRs produced by *M. sativa*.

In another IRLC legume, *Astragalus sinicus*, that forms indeterminate nodules with *Mesorhizobium huakuii* 7653R, Wei et al. (2022) showed that AsNCRs are downregulated in response to an *M. huakuii bacA* knockout mutant. The vast majority of AsNCRs (92.3%) in Group A 4 cysteine motif were downregulated more than 2-fold during symbiosis with the *bacA* knockout strain. Approximately 8.5% of AsNCRs in group B, 6 cysteine motifs were downregulated more than 10-fold. When testing the membrane permeability of *M. haukuii* treated with AsNCR100 peptides, they found a concentration-dependent increase in permeability. A surprising effect of AsNCR067 (group A, anionic) treatment was an increase in colony-forming units, interpreted as growth enhancement of *M. haukuii*. To identify bacterial targets of AsNCRs, a bacterial two-hybrid system (B2H) was used with AsNCR067 and AsNCR076 as bait. B2H identified chaperonins groEL1 and groEL3 as interactors with both AsNCRs. In parallel with the BacA transport system, the *E. meliloti* YeABEF transporter system contributes to MtNCR uptake into bacteroid cells. In knockout mutants, BacA can compensate for the absence of YeABEF, but the inverse is not true. Different mutants in the YeABEF system display different MtNCR sensitivity profiles, implying that the periplasmic binding protein YejA only interacts with a subset of MtNCRs (Nicoud et al., 2021).

## 4 Evolutionary genomics and gene family dynamics

### 4.1 Evolution of NCR genes within the IRLC

The expanded understanding of the *M. truncatula* NCR gene family has provided opportunities to conduct comparative analysis across legume species from gene and peptide perspectives. A high-quality genome of *P. sativum L.* revealed 360 PsNCR genes. Of these genes, 154 were novel, and the remaining had been documented in other legumes. *P. sativum* NCRs primarily follow the same pattern as NCRs in *M. truncatula*; group A has 4 cysteines, group B has 6 cysteines, and are composed of two exons separated by one intron. *P. sativum* also had NCRs that contained a third exon that codes for the last few amino acids of the mature peptide. Homologs of NCRs from *Cicer arietinum, T. pratense*, and *V. faba* could not be identified in *P. sativum*, which the authors claim supports independent evolution of NCRs in multiple species of legumes. The authors report that the signaling peptide is under stabilizing selection in contrast to the mature peptides, which are under neutral selection within *P. sativum*. When comparing *P. sativum* to other pea cultivars, the mature peptide was under diversifying selection (Zorin et al., 2022). The nodule proteome of *P. sativum* and *Lens culinaris* (lentil) was compared when inoculated with the same bacteria, *Rhizobium leguminosarum* UPM791. A total of 52 PsNCRs in the pea proteome and 65 LcNCR peptides were identified in the lentil proteome. These peptides were not conserved between the two legume species that diverged ~11.3 MYA (Kumar et al., 2016, p. 20). The comparison between these proteomes indicates a host-specific response to symbionts and suggests a parallel evolution of NCRs in multiple legume species, but also gene duplication and further evolution within a legume species (Durán et al., 2021). Additional support for the evolution of NCRs from defensins comes from a phylogenetic analysis of NCRs compared to actinorhizal nodule-specific defensins (ANDs), which are shown to be in the same monophyletic subclade. Supporting study proposed that nodule-specific defensins are in fact present throughout nodulating plants and that NCRs were lost in most lineages instead of convergently evolving in several (Salgado et al., 2022).

### 4.2 Evolution of NCR genes beyond the IRLC

Outside of the IRLC, there are no canonical NCRs; however, in the *Aeschynomene* genus (Dalbergioids), the previously described NCR-like peptides with Groups B and C cysteine motifs can induce a more efficient spherical nodule, similar to what is seen in the elongated indeterminate nodules (Lamouche et al., 2019). In contrast, *Arachis hypogaea* (peanut) produces defensin-like peptides with the Group B 6 cysteine motif and shares a conserved signal peptide with cysteine-rich secretory proteins (CAPs) that are both expressed late in nodulation. Due to the presence of Group B 6 cysteine motif and the lack of Group A 4 cysteine motif in *A. hypogaea*, there is evidence to support lineage specific evolution of a defensin-like outside of the IRLC (Raul et al., 2022). In a study of lineage-specific expanded (LSE) gene families, Trujillo et al. (2019) identified small signaling peptide gene families that are associated with nodulation that all have undergone LSE, including NCRs, LEEDS…PEEDS, *Aeschynomene* NCR-like peptides, and calmodulin-like proteins. A notable expansion was in diploid peanut *Arachis duranensis*, where cysteine-rich peptides related to antimicrobial peptide maize basic peptide (MBP-1) expanded to over 100 members and were upregulated in roots and nodules. This expansion supports the understanding that antimicrobial peptides and derivatives are used to control bacterial symbionts across many host species and can originate from different AMP gene families.

### 4.3 Coevolutionary dynamics and conflict

Biotic interactions exert strong and ongoing selection pressures that have long been believed to drive molecular evolution both within and between populations (Van Valen, 1973; Brockhurst et al., 2014). The high degree of diversity and rapid gene family expansion and turnover of NCRs and other nodule-expressed small signaling peptides (Kitaeva et al., 2021; Zorin et al., 2022) is consistent with coevolutionary balancing of antagonistic and cooperative dynamics between hosts and symbionts (Paterson et al., 2010; Montiel et al., 2017). Conflict between leguminous hosts and rhizobia may arise in many aspects of the relationship as a consequence of differences in optimal expression of traits (Friesen, 2012); such documented conflicts include host preferences for low or medium nodule numbers versus rhizobial preference for high levels of nodulation (Quides et al., 2021) and terminal differentiation of bacteroids that enhances nitrogen fixation but eliminates rhizobial reproduction (Alunni and Gourion, 2016). NCRs allow greater host control over mutualistic function by both limiting microbial exploitation of host resources and further optimizing symbiotic functioning to improve host benefits (Montiel et al., 2017; Pan and Wang, 2017). The diversification of NCRs and their induction of terminal differentiation of bacteroids is consistent with partner conflict over nitrogen fixation rate preferences; while a host may benefit from higher fixed nitrogen production that results from terminal bacteroid differentiation, the rhizobia suffer from higher metabolic costs and lost reproductive abilities (Alunni and Gourion, 2016). These coevolutionary dynamics manifest through the numerous molecular mechanisms of NCRs as antimicrobial weapons and facilitators of mutualism.

## 5 Functional studies and applications

### 5.1 NCR mechanisms of action

The “working balance” hypothesis provides a key framework for understanding NCR mechanisms in symbiosis (Pan and Wang, 2017). This hypothesis suggests that moderate NCR activity optimizes the mutualistic relationship, while excessive or insufficient NCR levels can harm both partners. NCRs achieve this balance through diverse mechanisms that go beyond simple antimicrobial killing.

Unlike some antibiotics, AMPs have a broad range of actions and can both be a direct method of killing pathogens or act as a delivery system for other compounds that cannot normally cross the membrane of microbial targets (Agbale et al., 2019). Evidence supports a direct interaction with bacterial membranes that can cause degradation through multiple mechanisms that all begin with binding and aggregating on the bacterial membrane (Mikuláss et al., 2016; Farkas et al., 2017). NCR peptides’ modes of action do not appear to be redundant, despite the large gene family size. NCRs are understood to interact at the membrane of bacterial and fungal cells, but there is additional evidence that many have a secondary intracellular site where they facilitate symbiosis.

The diversity of NCR mechanisms presents both opportunities and challenges for biotechnological applications (Lima et al., 2020). Many studies focus on the therapeutic potential of NCRs due to their short sequence, relatively low Minimum Inhibitory Concentration (MIC), and low toxicity to human cells; recent studies have largely focused on bacterial and fungal pathogens in both plants and humans (Table 3). Unlike single-target antibiotics, the multi-target nature of NCRs reduces the likelihood of resistance development but complicates structure–activity relationship studies and standardization of therapeutic doses. The working balance framework as applied to symbiotic studies may also apply to pathogen control, where optimal antimicrobial activity requires precise peptide concentrations and combinations rather than maximum doses (Pan and Wang, 2017; Pan, 2020).

**Table 3:** NCR-pathogen assays.

NCR ID	Pathogen (host)	Result	Reference
104 synthetic NCRs	ESKAPE*, *Acinetobacter baumannii, Candida albicans* (human)	NCR073, NCR336 and NCR358 was the most active peptide capable of killing all tested bacteria. *E. faecalis* was most resistant to NCRs, and *P. aeruginosa* was the most sensitive.	Zorin et al. (2022)
MtDef4 derivative	*Sclerotinia sclerotiorum* (Lib.) de Bary (*Glycine max* L. Merrill)	Binds to the pathogen cell wall and then permeates, spray application confers protection against the pathogen, with no phytotoxicity	Djami-Tchatchou et al. (2023)
NCR044	*Fusarium* spp. and *Botryis cinerea* (plant)	Inhibited growth of all, permeated fungal membranes, inhibited spore germination	Velivelli et al. (2020)
NCR169 and derivatives	*Escherichia coli* K-12 (human), *Ensifer meliloti* (symbiotic partner)	AMP activity against both *E. coli* was more sensitive. Membrane interaction facilitated by the cationic Lys-rich region	Isozumi et al. (2021)
NCR169C derivatives	ESKAPE*, *Listeria monocytogenes, Salmonella enterica* (human)	Killed ESKAPE and *L. monocytogenes, S. enterica*. Replacing cysteine residues did not improve AMP activity, but sometimes enhanced it. No hemolysis at AMP concentrations.	Howan et al. (2023)
NCR247	*H. influenzae, Porphyromonas gingivalis, Toxoplasma gondii* (human)	Prevented the growth of both bacteria, reduced plaque formation of *T. gondii,* and had negligible cytotoxicity in mammalian cell culture	Sankari et al. (2022)
NCR247 chimeras	ESKAPE*, *Listeria monocytogenes*, *Salmonella enterica* (human)	Killed all microbes tested at 25 uM or lower except *K. pneumoniae* and *L. monocytogenes*, antibiotic activity in C term, and requires a positive charge	Jenei et al. (2020)
NCR247, NCR335	*E. coli, B. subtilis, and S. cerevisiae* (human)	Membrane permeabilization and disruption, cell lysis	Farkas et al. (2018)
NCR247, NCR335	*Listeria* and *Salmonella* (human)	Killed in culture, membrane permeabilization and disruption, cell lysis	Farkas et al. (2017)

NCRs have been tested for antimicrobial activity against plant and human pathogens. Here is a summary of studies using NCRs and NCR derivatives against a variety of bacterial and fungal pathogens. *ESKAPE: *Enterococcus faecalis, Staphylococcus aureus, Klebsiella pneumoniae, Acinetobacter baumannii, Pseudomonas aeruginosa*, and *Escherichia coli*.

### 5.2 NCRs for plant pathogen control

Several NCRs have been assessed for their antimicrobial activity against plant fungal and bacterial pathogens (Tiricz et al., 2013). Velivelli et al. (2020) comprehensively analyzed MtNCR044 antimicrobial activity and found that it inhibited the growth of *Botrytis cinerea* (gray mold disease), *Fusarium graminearum* (Fusarium head blight of wheat and barley), *Fusarium oxysporum* (Fusarium wilt), and *Fusarium virguliforme* (sudden death syndrome of soybean). When *B. cinerea* germlings were challenged with recombinant MtNCR044, they showed signs of membrane permeabilization within 30 min post-treatment, and permeabilization peaking at 120 min post-treatment. When *B. cinerea* spores were challenged with MtNCR044, the membrane permeabilization was insufficient to kill the spores but successful in inhibiting their germination. Reactive oxygen species (ROS) production is a common consequence of antimicrobial peptide treatments and is frequently investigated when searching for mechanistic clues. ROS production increased in *B. cinerea* germlings in a time- and dose-dependent manner to levels sufficient to induce oxidative stress in the germlings. In contrast, no ROS production or change was observed in *B. cinerea* spores. The authors demonstrated that MtNCR044 can bind to multiple plasma membrane phospholipids, similar to the ancestral defensin-like peptides, and diffuse to the nucleoli of *B. cinerea* germlings after internalization. MtNCR044 treatment on lettuce leaves reduced gray mold disease lesion size, and on rose petals reduced the virulence of the disease. In a greenhouse assay, MtNCR044 was applied as a spray to *Nicotiana benthamiana* (tobacco) and *Solanum lycopersicum* (tomato) plants and was able to reduce gray mold disease severity for both plant species (Velivelli et al., 2020).

Huanglongbing (HLB), or the citrus greening disease, is caused by bacteria in the genus *Liberibacter* and includes ‘*Candidatus* Liberibacter asiaticus’ (*C*Las), ‘*Ca.* Liberibacter americanus’, and ‘*Ca.* Liberibacter africanus’ (da Graça et al., 2022). The causal bacteria are obligate intracellular pathogens vectored between citrus plants by two insects, the Asian citrus psyllid *Diaphorina citri* Kuwayama (Hemiptera: Liviidae) and the African citrus psyllid, *Trioza erytreae* del Guercio. *C*Las infection is limited to phloem in the plant and requires circulation through the insect vector before being deposited in phloem when the insect is feeding on the sap. *C*Las has orthologous genes to the pSymA plasmid and chromosomal genes of *E. meliloti*, prompting researchers to test the susceptibility of *C*Las to NCR peptides. Using an *in vitro* growth assay, *Liberibacter crescens* strain BT-1 (the closest culturable relative of *C*Las) was challenged by MtNCR peptide 20-mer fragments. In culture, growth inhibition of CLas up to 73% was observed. When testing the peptide fragments against *D. citri* adults, five fragments blocked the development of insects with more than 100 *C*Las cells, thus preventing high-titer, vector competent insects. Additionally, two peptide fragments were insecticidal to *D. citri* nymphs, suggesting that MtNCRs could be used to target multiple stages of the development and transfer of HLB bacteria (Higgins et al., 2024).

### 5.3 NCRs for human pathogen control

Development of AMPs into therapies targeting antimicrobial-resistant human pathogens has led to a search for naturally occurring AMP reservoirs. NCRs are good candidates for peptide therapies due to their small size, multiple AMP mechanisms, and low human cell toxicity. NCRs and NCR derivatives have contributed to our overall understanding of the structural or chemical source of antimicrobial activity and the mechanisms by which they kill or arrest microbial growth. A focus of antimicrobial studies is the ESKAPE pathogens, which refer to a set of human pathogens that have developed multidrug resistance: *Enterococcus faecium*, *Staphylococcus aureus*, *Klebsiella pneumoniae*, *Acinetobacter baumannii*, *Pseudomonas aeruginosa*, and *Enterobacter* spp. (Miller and Arias, 2024).

Recent studies highlight the diverse pathogens that NCRs can kill or inhibit, such as methicillin-resistant *S. aureus* (MRSA) and *K. pneumoniae* (Alhhazmi et al., 2024). A MtNCR247 chimera was tested against the ESKAPE human pathogens and showed antimicrobial action comparable to third-generation antibiotics with no toxicity against human cells (Jenei et al., 2020). MtNCR169 also binds to anionic cell membrane components and has antimicrobial activity against *E. coli* (Isozumi et al., 2021). MtNCR335 and MtNCR169 were active against *Candida* spp. and did not harm vaginal epithelial cells at antimicrobial concentrations. When split, the resulting C-terminal and N-terminal fragments were still antimicrobial, with the N-terminal showing the broadest activity against *Candida* spp. The C-terminal fragments reduced hyphae formation. All five fragments tested reduced biofilm formation, with sensitivity varying by *Candida* spp. These same fragments lacked cytotoxicity toward human keratinocyte HaCaT cells (Szerencsés et al., 2021). A new mechanism of action for MtNCRs was identified when Sankari et al. (2022) discovered that MtNCR247 binds heme 1:1. Heme and iron availability are key factors of the parasitic or pathogenic lifestyle of heme auxotrophs. When tested against *Haemophilus influenzae* (infections) and *Porphyromonas gingivalis* (periodontal disease), known heme auxotrophs, MtNCR247 killed both pathogens. Application of MtNCR247 to human foreskin cell cultures reduced the plaque formation of the parasite *Toxoplasma gondii* (toxoplasmosis). MtNCR247 was able to sequester heme from human blood, a positive sign that this NCR could be incorporated into treatments for diseases that release toxic amounts of heme into plasma.

### 5.4 Current limitations for NCR biotechnological applications

Despite promising initial results, several bottlenecks limit the agricultural implementation of NCRs as biopesticides. Peptide stability under field conditions remains a primary concern, as NCRs are susceptible to degradation by plant and microbial proteases, UV radiation, and extreme pH conditions (Al Musaimi et al., 2022). Delivery mechanisms also require optimization. Foliar spray applications have shown efficacy in controlled environments for certain plant species, but achieving consistent bioavailability and penetration to infection sites under variable field conditions and for a variety of plant physiologies remains challenging (Bechinger and Gorr, 2017; Preininger et al., 2018). Encapsulation technologies, such as nanoparticle delivery systems and polymer-based formulations, are being explored to enhance peptide stability and targeted delivery, drawing from advances in pharmaceutical peptide therapeutics (Abdulazizu et al., 2025). Beyond issues of delivery and deployment, applications of NCRs will inevitably need to deal with biological problems like the development of resistance in target organisms and identifying peptides with sufficient specificity (Magana et al., 2020).

The challenges facing NCR applications reflect the broader issues in the field of antimicrobial peptide applications. As with NCRs, AMP clinical therapy development has been hindered by difficulties in large-scale production, susceptibility to environmental conditions leading to degradation, specificity issues, and the general high costs of production (Biswaro et al., 2018). Fortunately, successes in AMP development provide valuable precedents and methodological frameworks for future NCR applications. Researchers have observed that AMPs exhibit pharmacodynamic properties that reduce the evolution of resistance by target organisms relative to traditional antibiotics, including rapid killing kinetics and narrow mutant selection windows (Lazzaro et al., 2020). Furthermore, technological advancements have been made to enhance endogenous AMP production, while the development of peptidomimetics—synthetic molecules that mimic peptide structures and features—offers improved stability and bioavailability (Sierra and Viñas, 2021). The successful translation of NCRs for field applications will require integrated approaches combining improvements in peptide design, delivery, and production systems informed by prior successes with AMPs.

## 6 Future directions

### 6.1 Basic research priorities

While much progress has been made recently in understanding the biology and evolution of NCRs in legumes, several open questions deserve further study. We still lack a predictive framework connecting the biochemical properties of specific NCRs to their effects on rhizobia in symbiosis. Furthermore, the relationships between NCR number and diversity and their functionality *in planta* are intriguing but not well-explained. The connection between temporal and tissue-specific expression with nitrogen-status signaling and potential NCR regulation of symbiotic nitrogen-fixation and nutrient exchange is worthy of further study. In addition, we still know relatively little about the downstream effects of NCRs in host-symbiont molecular pathways. The potential antagonistic coevolutionary dynamics between host and symbiont, as reflected in the ‘working balance hypothesis,’ deserve further investigation, as this could be a driving force behind the gene family expansion (Pan and Wang, 2017; Wendlandt et al., 2021). Finally, the evolutionary origins of NCRs and NCR-like peptides across legumes and the processes driving expansion and turnover of these gene families warrant additional study.

### 6.2 Applied research and translational opportunities

From an applied perspective, NCRs present a wide range of antimicrobial properties that could be harnessed to address antimicrobial-resistant pathogens in both plant and animal systems. NCRs are short, stable, and demonstrate antimicrobial action against plant and human pathogens from diverse branches of life *in vivo* and *in vitro*. Current research supports the use of plant AMPs for crop protection, post-harvest treatment, and safe food storage (Heymich et al., 2021; Liu et al., 2021; Taveira et al., 2022). Natural and synthetic AMPs to treat human infections are in multiple phases of clinical trials (Moretta et al., 2021). As we continue to search for tools to address crop loss from plant pathogens, food loss post-harvest, and antibiotic resistance, we need to expand our search for AMPs outside of model species. In parallel, teasing out the mode of action of the best candidate AMPs is necessary to take advantage of the mechanisms that exist in combination, reducing the chances that pathogenic microbes will develop resistance. AMPs like NCRs, whose role in nature is to control coevolving bacterial symbionts, are an excellent testbed for effective microbial control; future study could expand the range of NCRs tested as well as explore the optimal delivery methods to make use of these in practice.
